# Trigeminal Neuralgia – rethinking the “suicide disease” label

**DOI:** 10.1192/j.eurpsy.2025.1896

**Published:** 2025-08-26

**Authors:** R. Neto, B. Fonseca Silva, M. Remelhe, R. Araujo

**Affiliations:** 1 ULSGE, Vila Nova de Gaia, Portugal

## Abstract

**Introduction:**

Trigeminal Neuralgia (TN) is a rare condition characterized by recurrent brief episodes of unilateral and excruciating facial pain, typically triggered by innocuous stimuli. It is historically known as the “suicide disease”, emphasizing the severity of the attacks and its impact on patients’ mental health.

**Objectives:**

We aim to highlight the risk of psychiatric comorbidities following TN diagnosis and discuss the potential burden depicted by the phrase “suicide disease”.

**Methods:**

We presented a case report and conducted a non-systematic review of the literature.

**Results:**

A 72-year-old female patient with history of diabetes, hypertension, dyslipidemia, recurrent thrombophlebitis but no previous relevant psychiatric history, presents with depressive mood, anhedonia, insomnia, reduced appetite and feelings of hopelessness, which began 3 months prior. These symptoms started shortly after she began experiencing paroxysms of intense electric shock-like pain in the right hemiface, allodynia (specially triggered by the wind, talking, chewing and light touch) and lacrimation of the right eye. The patient had multiple consultations with neurology and psychiatry physicians. TN was presumed and the patient initiated treatment with pregabalin and intravenous infusions of lidocaine, as well as antidepressants. Magnetic resonance angiography revealed neurovascular compression of the right trigeminal nerve, supporting the diagnosis. Depressive symptoms aggravated and she experienced recurrent suicidal thoughts as she became aware of the TN diagnosis and experienced debilitating symptoms due to initial suboptimal pain relief. Oxcarbazepine was later introduced in the treatment plan and pain relief was slowly achieved. Suicidal ideation waned despite maintenance of depressive mood. Evidence shows there is a higher risk of newly diagnosed depressive, anxious and sleep disorders following TN diagnosis, most likely due to its deleterious effect at a psychological, behavioral and social level. Currently, however, the phrase “suicide disease” may be an ill-suited one as the lack of information on suicide rates among patients with TN and the availability of new and more efficient therapeutic options do not support its present use.

**Conclusions:**

This case exemplifies the increased risk of new psychiatric comorbidities following TN diagnosis, further aggravating patients’ quality of life. Despite its historical significance, the label “suicide disease” seems to lack current applicability and may not only harm patients’ understanding and acceptance of the diagnosis, but also exacerbate fear and stress concerning its prognosis.

**Disclosure of Interest:**

None Declared

